# Case Report: Deprescribing psychotropic medication with an adolescent boy with complex emotional and behavioural challenges

**DOI:** 10.3389/frcha.2026.1808598

**Published:** 2026-04-20

**Authors:** Laura Theall, Serena Atallah, Ajit Ninan, Kim Arbeau, Alicia McLean

**Affiliations:** 1 Child and Parent Resource Institute, London, ON, Canada; 2 Department of Social Work, University of Windsor, Windsor, ON, Canada; 3 Division of Child and Adolescent Psychiatry, Western University, London, ON, Canada; 4 Catholic Children's Aid Society, Toronto, ON, Canada

**Keywords:** case report, children, complex needs, deprescribing, psychotropic medication

## Abstract

**Background:**

The prescription of psychotropic drugs to children and youth has been on the rise. Polypharmacy—the act of prescribing multiple medications—is a risk for children and youth with emotional and behavioural challenges, as prescribers may administer multiple medications to treat persistent symptoms or the side effects of other medications without solid evidence. The use of psychotropic medications with children and youth has been linked to several negative health outcomes, and further risks exist in cases of polypharmacy such as increased and more severe medication side effects, toxicity, and non-compliance.

**Objective:**

This case report shares the treatment approach of medication deprescribing coupled with attachment-focused psychosocial intervention for managing emotional/behavioural challenges.

**Case presentation:**

An adolescent boy in foster care experiencing persistent behavioural disturbance, neurodevelopmental disorders, and attachment disruption presented for treatment at a tertiary care agency for children and youth with complex combinations of mental health, developmental, and behavioural needs having been prescribed five psychotropic medications. By the end of his treatment, he was on no medications and had shown behavioural gains (more co-operative, calmer, reduced impulsivity, aggression, and irritability) with no concerning changes in symptoms.

**Conclusions:**

The current case serves as an example for clinicians to critically consider the risks and benefits of patients' medication regimen and the value of deprescribing with psychosocial supports to manage symptoms.

## Introduction

Psychotropic medications have become a dominant component in psychiatric treatment plans for children and youth with complex presentations. Prevalence of psychotropic medication prescriptions for children and youth have increased over time (1–4). For example, in Canada prescriptions increased by nearly 7% between 2008 and 2015 in the province of Alberta (1) and 3.8-fold from 1996 to 2011 in British Columbia (2). This is the case even though many psychotropic medications are often prescribed off-label, meaning they have not been approved for use with children and youth for the condition being treated (5), and often without associated psychotherapy to help with symptom management (6). The use of psychotropic medication for children and youth has been linked to a myriad of negative health outcomes, including cardiotoxicity, suicide, somatic symptoms such as gastrointestinal issues or headaches, metabolic effects (e.g., weight gain, changes in blood pressure and cholesterol, glucose intolerance), and movement disorders (e.g., tremors, rigidity) (7, 8). The presence of side effects can lead to a prescribing cascade of additional medications (9, 10). Polypharmacy (prescription of multiple medications) has also increased, with highest rates disproportionately amongst children in the foster care system (11–13). Further risks exist in cases of polypharmacy, such as increased and more severe medication side effects and toxicity (14).

Relying on psychotropics to treat behavioural issues in children and youth has been debated as some are concerned that medication is used to replace psychosocial interventions (15). Early childhood experiences of chronic stress due to neglect, emotional/verbal abuse and/or physical abuse, can lead to behavioural dysregulation that can mimic criteria for neurobiological diagnoses such as attention deficit hyperactivity disorder (ADHD), oppositional defiant disorder (ODD), anxiety and conduct disorders (16). According to attachment theory, children develop emotion regulation and relational skills through repeated interactions with a positive, responsive, and reliable caregiver (17). Adverse experiences with unreliable or harmful parenting disrupts a child's attachment patterns which impacts their capacity to regulate their own emotions and interferes with their ability to form healthy relationships with caregivers and others throughout development and potentially into adulthood (18). Children with fetal alcohol spectrum disorder (FASD) in particular experience high rates of early adversity, inconsistent caregiving, and foster placements which increase their risk for disrupted attachment (19). Treating resulting behaviours with medications alone without attending to the child's underlying disrupted attachment patterns and maladaptive coping strategies may not produce lasting therapeutic change (20). Attachment-focussed interventions utilize attachment-based models of understanding emotional and social challenges to foster the growth of healthier attachment patterns, leading to improved functioning in these areas.

Consideration for which symptoms require medication to manage vs. which should be addressed with psychosocial supports is vital for best practice care. Deprescribing involves the systematic identification and discontinuation of medications when the harms of the medication outweigh the benefits (21, 22) or when the medication is deemed inappropriate (23). There are few published examples of deprescribing as a viable solution that can result in a maintenance or reduction of symptoms and adverse effects for children and youth with complex conditions that require treatment (24). The current case report aims to add to the evidence in this area by demonstrating positive outcomes of deprescribing with a patient presenting with a combination of behavioural challenges, neurodevelopmental differences, attachment disruption and polypharmacy.

## Case description

Jeffrey (name changed to a pseudonym) was a 12-year-old boy of African Canadian descent in foster care who was referred to outpatient services at a tertiary centre for children and youth with complex combinations of mental health, behavioural, and developmental needs. His Children's Aid Society (CAS) guardian provided written informed consent to participate in this case report with Jeffrey's assent. CAS is a non-profit organization in Canada mandated to protect the welfare of children and youth. Jeffrey was initially referred for a diagnostic opinion and medication review by his treating psychiatrist. Priority concerns were episodes of behaviour dysregulation and challenges with impulse control. During such episodes, Jeffrey's behaviour escalated from verbal to physical aggression including property destruction and self-injurious behaviours, often endorsing hopelessness and suicidal and homicidal ideation. The intensity of behaviours repeatedly involved police intervention. Episodes lasted up to an hour, occurring about three to four times per week. Jeffrey also exhibited social deficits such as antagonizing others using known sensitivities, nonadherence to expectations, argumentativeness around most tasks, difficulties with organization and forgetfulness, distractibility, rushing through schoolwork, making careless mistakes, and jumping from one activity to another without completing the previous task. These behaviours existed despite being medicated for ADHD. Jeffrey's baseline mood state was described as irritable. Upon referral to the centre, the interRAI™ Child and Youth Mental Health (ChYMH) instrument was completed by a trained assessor (25). Jeffrey scored in the very high range on the externalizing scale, very high on the disruptive aggressive behaviour scale, and high on the hyperactive/ distractibility scale; internalizing and anxiety symptoms were in the low/moderate range, and there was no current sleep disruption (26–29). Individual ChYMH item scores indicated that episodes of impulsivity, verbal abuse, outbursts of anger, disruptive and destructive behaviour were exhibited multiple times daily. At the time of assessment, Jeffrey was on the following medications: melatonin (3 mg orally at bedtime), olanzapine (2.5 mg orally twice daily and 5 mg orally at noon; also prn olanzapine ODT, fast dissolving, 5 mg orally once daily as needed for agitation), quetiapine (25 mg orally 2 times daily), guanfacine extended release (4 mg orally in the morning), and methylphenidate HCl extended release (18 mg orally in the morning). The sequence by which the medications were originally prescribed was unknown. Jeffrey held diagnoses of a learning disorder (reading, written expression, math), ADHD (combined type), an intellectual disability (which was subsequently refuted), ODD, and FASD without sentinel facial features.

### Developmental and social history

Alcohol and cigarettes were used during pregnancy. With respect to milestones, at 1.5 years old, Jeffrey showed no speech sounds and presented similar to a 10 to 13-month infant and had some developmental delays in fine and gross motor skills. Behavioural concerns emerged in junior kindergarten (age 4), including defiance, disruptiveness that required 1:1 support to follow expectations, and not taking responsibility for actions continuing over time, in addition to academic difficulties. At age 6, concerns were noted for speech articulation. Jeffrey was first apprehended by CAS at age 5 for domestic violence and parental substance use. He was in and out of placements with his biological family, kinship placements, and multiple foster care placements, before being placed in a group home and eventually with two CAS supported caregivers. In his early years Jeffrey showed evidence of a global developmental delay and high levels of aggression, destruction, and disobedience. With Jeffrey's history of early childhood adversity, placement instability, and longstanding challenges with emotion regulation and social navigation, it was suspected that he experienced significant disruption to attachment development. Jeffrey had a learning disorder, and experienced patterns of inattention, hyperactivity, and impulsivity. Experiencing these challenges was likely a daily source of frustration for Jeffrey, with impulsivity specifically equating to a tendency to be reactive to states of frustration leading to the development of maladaptive coping strategies which became entrenched over time. Jeffrey had been involved in several day treatment centres and outpatient supports, counselling, check-ins with his CAS guardian, and had access to a therapy dog. These interventions demonstrated varying degrees of effectiveness that were time limited.

### Service goals, treatment process, and outcomes

Upon admission to the tertiary agency, Jeffrey was connected with a psychiatrist who oversaw a medication review following diagnostic confirmation. Jeffrey's diagnoses were affirmed for FASD without sentinel facial features, ODD, and learning disorder with specific impairments in reading, writing, and math, with addition of disturbance of attachment. The ADHD diagnosis was retained at the start of treatment, and there was a rule out of disruptive mood dysregulation disorder. The psychiatrist made an internal referral for Jeffrey and his caregivers to receive psychosocial supports from a multi-disciplinary attachment team at the agency, which provides trauma-informed assessment, consultation and education to support children and youth (up to age 18) with a suspected or confirmed attachment disruption. Jeffrey's foster parents attended an online parenting program delivered by the team's trained therapists called eConnect for kinship & foster parents (30), an evidence-based trauma-informed 10-session program delivered virtually, focused on supporting foster parents to strengthen attachment relationships with adolescents in their care who have experienced early negative life events. The team additionally provided follow up consultation to caregivers with a behavioural consultant in order to solidify the understanding and utilization of this psychosocial intervention. While receiving tertiary services, Jeffrey also continued to receive counselling from a community service provider.

**Figure 1:**
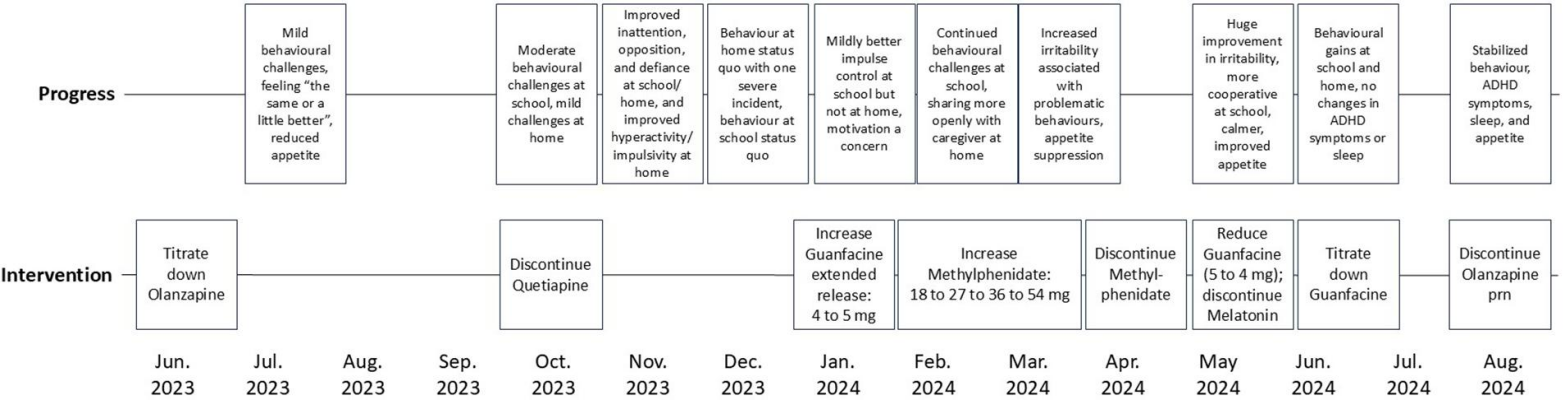
Episodes of care timeline.

The service goal at the tertiary agency collaboratively developed by the psychiatrist, Jeffrey and his caregivers was to review Jeffrey's medications and determine the necessity and benefits of each. The initial request to come off medications came from Jeffrey, which was supported by his caregivers, CAS worker, and service team. The agency's approach to deprescribing has been described by Theall and colleagues (22), which includes conducting a review of current medications for indication and risk/benefit, employing a stepwise approach with attention to proper timing and setting/environmental considerations, while partnering with the child/youth and caregivers and ensuring careful monitoring for improvements and adverse effects, with continuity of clinical oversight and appropriate psychosocial supports. The episodes of care timeline for Jeffrey's case is provided in Figure 1.

Given the redundancy of two antipsychotics (olanzapine and quetiapine) and concerns regarding the side effect burden, the first candidate medication for deprescription was olanzapine (5 mg) which was reduced by 50% (2.5 mg) every two weeks over 4 weeks. After deprescribing the olanzapine, Jeffrey indicated he was feeling “the same or a little better” and his appetite had reduced, which was viewed positively as he previously had a high appetite for less nutritional food. He experienced mild incidents of non-cooperative behaviour and no incidents of destructive or aggressive behaviour during this stage.

Given the positive outcome after deprescribing olanzapine, the benefit of quetiapine was assessed by trialing a discontinuation. As Jeffrey was transitioning into middle school, his quetiapine dose was not modified until after his first month of school. After discontinuing quetiapine over 2 weeks (100% morning dose discontinued first, followed by 100% discontinuation of the afternoon dose one week later), Jeffrey exhibited continued behavioural challenges at school and mild challenges at home, but no concerning changes overall. After repeating ADHD rating scales following discontinuation of olanzapine and quetiapine, inattention at school and home, hyperactivity and impulsivity at home, and opposition/defiance at home and school improved. Despite eliminating two antipsychotic medications, Jeffrey demonstrated improvements in his intermittent behavioural concerns, inattention, and baseline tendencies towards oppositionality/irritability.

The repeated ADHD rating scales indicated minor residual symptoms; therefore, a decision was made to modify Jeffrey's ADHD medications for optimal results, starting by increasing guanfacine extended release from 4 mg to 5 mg which resulted in mild improvements in impulse control and behaviours in the classroom. It was decided to incrementally increase methylphenidate HCl extended release from 18 mg to 36 mg to 54 mg, due to the potential for a more robust effect on ADHD symptoms. This was followed by a sharp rise in irritability associated with problematic behaviours, and appetite suppression. Jeffrey, his caregivers, and the psychiatrist concluded that the adverse effects of the methylphenidate outweighed the benefits, and a decision was made to discontinue the medication immediately without a taper schedule.

It was reported by Jeffrey and his caregivers that there was a “huge improvement” in irritability after discontinuing methylphenidate, suggesting that Jeffrey's irritability was an adverse side effect that had been masked throughout the time of use. At school he showed an improved ability to take responsibility for his behaviours, accept redirection and debrief situations, and was described as calmer. His caregivers noted he had longer periods of positive behaviours, improved baseline mood, and improved appetite. At this meeting, it was decided to reduce guanfacine extended release by 1 mg, back to 4 mg, given the lack of substantial improvement at the higher (5 mg) dose of guanfacine, and discontinue melatonin given an absence of sleep concerns. No changes in ADHD symptoms or sleep were noted subsequently.

As Jeffrey and his caregivers articulated his desire to come off all medications, it was decided to reduce guanfacine extended release (4 mg) over 8 weeks at a rate of 25% of the highest dose administered (i.e., reduction of 1 mg per 2 weeks). No symptom changes were noted after deprescribing the guanfacine. As Jeffrey had not needed his olanzapine prn in over a year, that prescription was also discontinued. Throughout the deprescribing period ongoing behavioural concerns were managed with psychosocial interventions. His caregivers were involved in the agency's attachment consultation and education program, which provided attachment-informed parenting approaches to help manage behavioural concerns related to attachment disruption.

Jeffrey was assessed once more after his transition into high school. He seemed to have had a temporary increase in problematic behaviours associated with the transition, and concerns still stemmed from his social navigation and decision-making skills, along with a baseline tendency towards oppositional behaviours. Jeffrey continued counselling that was in place through the community service provider, along with extra-curricular activity support and a special education teacher. Jeffrey had made gains overall in his behaviour with no long-term increases in concerning behaviour despite being completely off all medications. After demonstrating sustained improvement over several months without any prescribed psychotropic medications and optimizing psychosocial interventions, Jeffrey was discharged from tertiary services. An interRAI™ ChYMH assessment at discharge measured Jeffrey's treatment gains compared to his initial assessment: Externalizing symptoms reduced from the very high range to the moderate range; disruptive aggressive behaviour reduced from very high to low; and hyperactive/distractibility reduced from high to low (see Table 1). Impulsivity, verbal abuse, outbursts of anger, disruptive and destructive behaviour each decreased from being exhibited multiple times daily at intake, to no episodes in the days preceding the discharge assessment. His diagnosis of ADHD was removed by the time of his discharge from the agency, as his symptoms were shown to be mild and deemed better explained by the FASD diagnosis.

**Table 1:** Initial and discharge scores on select interRAI™ scales.

interRAI ChYMH scale	Initial raw score	Initial score category	Discharge raw score	Discharge score category
Externalizing	34	Very High	12	Moderate
Disruptive Aggressive Behaviour	18	Very High	3	Low
Hyperactive/ Distractibility	12	High	6	Low
Internalizing	3	Low	4	Low
Anxiety	5	Moderate	3	Moderate
Sleep	0	No Risk	0	No Risk

A follow-up found Jeffrey continuing in high school, living within a stable home environment with his caregivers and free from medications. While he did have an increase in challenging behaviours during his first year of high school, he and his caregivers continued to follow through on trauma-responsive psychosocial interventions to support his behaviours. His caregivers found it helpful to be reminded that as a teenager Jeffrey is going through hormonal changes, forming his own identity, testing boundaries, and experiencing other typical teen challenges that do not require medication treatment.

## Discussion

Jeffrey's case is an example of a successful medication deprescription approach. Access to psychosocial interventions along with the excellent support provided by his caregivers, CAS, and the school facilitated Jeffrey's deprescription. Wraparound care has been associated with reduced residential restrictiveness and improved outcomes in both educational and mental health domains (31), and psychosocial interventions have been shown to reduce aggression and improve social functioning in children and youth with disruptive behaviour disorders (32). Jeffrey's CAS guardian, a social worker, was especially instrumental in ensuring wraparound care. She met and communicated with Jeffrey and his caregivers on a regular basis to obtain day-to-day information on how Jeffrey was doing. She updated CAS management on Jeffrey's progress and advocated for his desire to be off medication. She facilitated conversations with his caregivers and the school around typical teenage behaviour to try and deter from over-pathologizing Jeffery's behaviours at times. The CAS guardian also encouraged Jeffrey to speak openly and honestly about what he felt he needed from the care team to help ensure his best interest was at the forefront. Having control to make meaningful choices in his own care may have contributed to Jeffrey's behavioural improvement. Self-determination and autonomy in adolescence have been found to reduce the likelihood of problem behaviour and support more adaptive emotional and behavioural development (33). Importantly, all individuals involved in Jeffrey's deprescribing plan were highly supportive, and Jeffrey demonstrated strong motivation to safely discontinue his medications in order to improve how he felt and functioned.

Evidence suggests that deprescribing is at risk when there is insufficient communication between physicians and patients/caregivers regarding the deprescribing plan (34). Attention to the timing of medication deprescription and proceeding with a stepwise approach in collaboration with the patient and caregivers contributed to successful deprescribing in the current case. By timing the reduction of medication so that it did not interfere with major life transitions (e.g., changing schools), the care team ensured that Jeffrey was not experiencing too many overwhelming changes simultaneously. By systematically reducing medications one-by-one while caregivers carefully monitored symptoms, the care team was able to observe medication-specific changes and respond accordingly. Vigilant tracking of Jeffrey's improvements and early detection of worsening symptoms were key to support his safety during the deprescribing period. His CAS guardian provided consistent oversight and clinical observations about his behaviours, while his foster parents carefully monitored target symptoms throughout the deprescribing period and shared their findings with the clinical team. Communication outside of scheduled appointments was encouraged as needed to ensure timely access to psychiatry support.

While the exact history of Jeffrey's prescribing cascade is unknown, certain aspects of Jeffrey's case may have increased the likelihood of psychotropic polypharmacy – specifically, having a FASD diagnosis (35) and being placed in foster care (13, 36). Children and youth with these profiles have a greater risk of exposure to adverse pre-and-post-natal experiences, which can be related to psychiatric complexity and comorbidity (37–39). Providing effective, person-centred care for children and youth with complex trauma histories requires shifting the focus from “What is wrong with the patient?” to “What has happened to the patient?” (40). A key aspect of Jeffrey's treatment was caregiver education on positive attachment-focused parenting, and his foster parents were highly receptive to strengthening supportive parenting strategies. This case report demonstrates that deprescribing in combination with psychosocial supports can be both appropriate and beneficial even when working with children and youth with significant psychiatric complexity.

## Limitations

There are limitations to the current case report to consider. First, findings represent a single case and therefore may not be applicable to the broader pediatric population. Still, the current paper contributes to the pediatric deprescribing literature to continue building an evidence base for this emerging practice. As clinician discomfort and lack of knowledge are cited barriers to deprescribing (9), more case examples similar to the current report and that of McLennan (24) are needed as well as support tools such as clinical guidelines, checklists, and professional education. Second, due to the non-experimental nature of this case report, a causal relation between medications and symptoms cannot be confirmed. Though, it is well established that psychotropic medications can lead to adverse side effects for children and youth (7, 8, 14). For example, stimulants—such as methylphenidate HCl extended release—have been known to produce irritability (15), as seen in the current case. Jeffrey's irritability likely exacerbated the behavioural challenges that his medications were prescribed to reduce. Indeed, when methylphenidate HCl extended release was increased, an increase in irritability and associated problem behaviours were observed, and when methylphenidate was deprescribed, a decrease in irritability and improved calmness and cooperativeness were evident. While causality cannot be confirmed, it serves as an example of how a patient's side effect profile might be exacerbating psychiatric challenges, and how symptoms could be reduced after deprescribing. Any identified adverse effects may warrant prioritization within the deprescribing plan.

## Conclusion

Thus far, the extant literature on deprescription has focused predominantly on older populations with a dearth of literature focused on deprescription amongst child and youth populations (21). This case offers an example of successfully deprescribing medications with an adolescent with complex needs. Although each patient will require an individualized approach, certain principles used in the current case could be applicable across cases, such as being mindful of deprescription timing, taking a systematic stepwise approach, clear and accessible communication between all individuals involved, taking into account patient preferences/values, partnership with caregivers, consensus in goals with support to achieve goals, and availability of individualized psychosocial interventions. Additionally, pharmacologic considerations that were used to decide which medication to deprescribe could be applied to other young patients, including: (a) the presence of two or more medications in the same category (e.g., antipsychotics) given the additive side effect burden and paucity of evidence to justify this; (b) optimization of one medication in attempt to eliminate another medication used to treat the same condition; (c) uncertainty of benefit; and (d) known adverse effects of a medication. Future research should continue to focus on studying the processes and benefits of medication deprescription in child and youth populations. With psychotropic medication prescription on the rise (41), clinicians are encouraged to consider the risks and benefits of patients' medication regimens throughout treatment, how psychosocial interventions might be used in place of medications, and how patients' side effect profiles might be exacerbating challenges. Both pharmacological and non-pharmacological interventions should undergo ongoing evaluation to ensure their continued effectiveness and relevance (23).

## Data Availability

The data analyzed in this study is subject to the following licenses/restrictions: The data supporting this case report cannot be shared due to patient privacy and confidentiality considerations. Requests to access these datasets should be directed to laura.theall-honey@ontario.ca.
